# Movable Cecum

**Published:** 1911-09

**Authors:** 


### Movable Cecum

Movable cecum (P. Duval, *Paris Médical,* June 3, 1911), occurs mostly in women (75 per cent.), and during adolescence (between the 15th and 25th year of life). The condition is evinced by three symptoms,—colic, constipation and typhlatony with typhlectasia. The pain element predominates; the pains belong to the chronic appendicitis type of pains. On palpation there is tenderness at McBurney's point. The acute pains are accompanied by vomiting, pallor, a sensation of sudden exhaustion, nausea, and occasionally by loss of consciousness. They often cease abruptly when the patient changes his position. The chronic pains provoke a nauseous condition, loss of appetite and an apathetic state resembling that in the wake of intestinal intoxication. The constipation is varying, irregular, is not influenced by diet nor by treatment in general, and is occasionally interrupted by diarrheal attacks. Typhlatony and typhlectasis are evidenced by an examination of the iliac fossa. In lean subjects there is occasionally seen a curved distension in the cecal region. This curve may be the seat of visible contractions; one may be able to take hold of it and thus be able to ascertain its contours. It feels elastic to the touch like an air pillow. On palpation a large cecum, gurgling under the fingers, can always be made out.—*Archives of Diagnosis.*

